# Family planning practices of women working in the Cambodian garment industry: a qualitative study

**DOI:** 10.1186/s40834-020-00116-7

**Published:** 2020-08-11

**Authors:** Chisato Masuda, Elisa Oreglia, Sokhey Ly, Megan McLaren, Caroline Free, Camille Tijamo, Amra Ou, Michelle Helena van Velthoven, Amy Williamson, Chris Smith

**Affiliations:** 1 School of Tropical Medicine and Global Health, Nagasaki University, Sakamoto 1-12-4, Nagasaki, 852-8523 Japan; 2 Kings College London, London, UK; 3 Marie Stopes International Cambodia, Phnom Penh, Cambodia; 4 Marie Stopes International, London, UK; 5 Department of Population Health, London School of Hygiene and Tropical Medicine, Keppel Street, London, WC1E7HT UK; 6 Clinical Research Department, London School of Hygiene and Tropical Medicine, Keppel Street, London, WC1E7HT UK

**Keywords:** Family planning services (mesh terms), Medical abortion, Contraception (mesh terms), Long-acting reversible contraception (mesh terms), Contraception, Barrier (mesh terms)

## Abstract

**Background:**

Women working in Cambodian garment factories have unmet needs for family planning (contraception and safe abortion) services, because of their background and living conditions. This study describes their experiences regarding abortion and contraception as part of a larger project to develop an intervention to support comprehensive post-abortion care.

**Methods:**

We conducted semi-structured interviews with women seeking abortion services at private health facilities. In addition, we interviewed the private providers of abortion and contraception services surrounding garment factories. Interviews lasted up to 60 min and were conducted in Khmer and later translated into English. A thematic analysis was undertaken, with medical abortion experiences coded according to the Cambodia comprehensive abortion care protocol.

**Results:**

We interviewed 16 women and 13 providers between August and November 2018. Most women reported being married and had at least one child. Among factory workers the major reported reasons for abortion were birth spacing and financial constraints. Family, friends, or co-workers were the major information resources regarding abortion and contraception, and their positive or negative experiences strongly influenced women’s attitude towards both. Medical abortion pills were not always provided with adequate instructions. Half of the participants had a manual vacuum aspiration procedure performed after medical abortion. While women knew the side effects of medical abortion, many did not know the adverse warning signs and the signs of abortion completion. Only three women started post abortion family planning, as most of the women expressed fear and hesitation due to concerns about side effects of modern contraception. Fear of infertility was particularly reported among young women without children.

**Conclusion:**

This research shows that in this setting not all women are receiving comprehensive abortion care and contraceptive counselling. Provision of accurate and adequate information about abortion methods and modern contraception was the dominant shortfall in abortion care. Future work to address this gap could involve the development of appropriate interventions and informative tools for women in the Cambodian garment industry such as through existing client contact-centres or social media, including creation of videos or posts on topics that come from clients questions.

## Plain English summary

Women working in Cambodian garment factories may have higher unmet need for family planning than other women in Cambodia. Most garment factory workers are living away from home and are aged under 30. Half have only primary school education. This study describes their experiences of contraception and abortion. We conducted interviews with 16 women seeking abortion services at private health facilities, and 13 private providers surrounding garment factories. Most women were married and had at least one child. Among factory workers the major reported reasons for abortion were birth spacing and financial constraints. Family, friends, or co-workers were the major information resources regarding abortion and contraception, and their positive or negative experiences strongly influenced women’s attitude towards both. Medical abortion was not always provided with adequate instructions. While women knew the side effects of medical abortion, many did not know the adverse warning signs and the signs of abortion completion. Only three women started contraception after abortion, as most of the women expressed fear and hesitation of real or perceived side effects associated with modern contraception. Fear of infertility was particularly reported among young women without children. This research shows that in this setting not all women are receiving comprehensive abortion care and contraceptive counselling. Provision of accurate and adequate information about abortion methods and modern contraception was the dominant shortfall in abortion care. Future work needs to address this gap by developing appropriate and effective interventions and informative tools for women in the Cambodian garment industry.

## Background

In 2017, an estimated 214 million women in low- and middle-income countries wanted to prevent pregnancy but were not using modern contraceptive methods. Of these women, 155 million used no method and 59 million used a traditional method. Estimates from global surveys indicate that yearly there could be 67 million fewer unintended pregnancies if all needs were met, thus preventing an estimated 36 million abortions and 76,000 maternal deaths [1]. In Cambodia, the use of modern contraception amongst currently married women rose from 19% in 2000 to 39% in 2014, however 18% of women were relying on traditional methods and 12% had an unmet need for family planning in 2014 [2]. More recent projection shows that about half of women using contraception choose short-acting modern methods, less than 25% of women use permanent or long-acting modern methods and slightly more than 25% rely on traditional methods [3]. Modern contraception is widely available in Cambodia, however, fear of side effects and health concerns have been identified as common reasons for non-use or discontinuation [2, 4, 5]. Perceived low risk of pregnancy, for example infrequent sexual activity due to the separation with partner, is another reason for not using contraception [4]. Thus, many Cambodian women remain at risk of unintended pregnancy.

Abortion was legalized in Cambodia in 1997 with the requirement that within the first trimester of pregnancy only qualified healthcare providers in certified health facilities can conduct abortions [6, 7]. Medical abortion, a combination of misoprostol and mifepristone, was approved in 2010 [7, 8], together with the dissemination of a national protocol for Comprehensive Abortion Care (CAC) that outlines the procedures to be followed by health providers to ensure safe abortion care [9]. Between 2010 and 2014, the proportion of medical abortions increased from 31 to 47%, while surgical abortion decreased from about 68 to 60%, and those using a traditional or unspecified method reduced from 3% to almost zero [2, 10]. Conversely, the proportion of women receiving help with abortion from a qualified health provider decreased from about 67% in 2010 to 60% in 2014 [2, 10]. These data indicate that medical abortion is increasing among Cambodian women and they are using it alone and at home. Home-based abortion is regarded as a safe method when adequate measures and support are in place [11]. However, the increasing rate of self-administration of medical abortion in Cambodia raises concerns on whether women are receiving quality comprehensive abortion services by health providers and can manage the process safely.

This study focuses on women working in a garment factory in the capital of Cambodia, Phnom Penh. Garment factories are the biggest manufacturing sector in Cambodia [12]. Roughly 800,000 people work in garment factories in Cambodia, of whom about 85% are female [13, 14]. They are potentially a vulnerable population for reproductive health issues; for example, around 70% are younger than 30 years, half of them have only a primary school education, and most have migrated from rural areas away from their family and community support [14]. According to a survey among female factory workers, they have a higher rate of abortion compared to young women in the national survey, whilst the use of contraception is comparable [2, 4, 15]. This implies that women working in garment factories have unmet needs for family planning (i.e. contraception and safe abortion) services compared with women in the general population [15, 16]. This paper aims to describe women’s experiences of abortion and contraception services as part of a larger project to develop an intervention to support comprehensive post-abortion care, which follows from our previous work in Cambodia [17–19].

## Methods

### Study design

The study was designed as a ground-up exploratory, qualitative study to discover themes and challenges that had not been unearthed by previous research. We thus conducted semi-structured interviews with women working in garment factories to find out about their abortion and contraceptive services seeking behaviour and experience. Additionally, we interviewed private providers of abortion and contraception services to understand the availability of services at the study site and their views on practices and demands for abortion and contraception. The study was a collaboration between academics from different disciplines (medicine, information science and linguistics) and the Non-Governmental Organisation (NGO) Marie Stopes International Cambodia.

### Study setting

This qualitative study was conducted from August to November 2018 in a suburb in Phnom Penh where many garment factories cluster. Table 1 provides an overview of the garment factory industry and Table 2 describes private providers of abortion and contraception in Cambodia.

**Table 1:** The garment factory industry in Cambodia

Cambodia’s garment sector employs about 85% of all Cambodian factory workers and provides 40% of the Gross Domestic Product. The minimum monthly wages of garment factory workers increased from 61 USD in 2012 to 170 USD in 2018 [13]. Workers typically work six days a week from 7 AM to 4 PM with an hour lunch break, unless there is overwork in the evening. They perform a specific task; for example, cutting fabric, stitching clothing, quality checks, or supervising other workers. Garment factory workers in Phnom Penh often share accommodation with relatives or friends within walking distance of the factory. Around the factory, there is usually a market and numerous small restaurants and shops selling commodities [15].	

*Image shows a garment factory work setting (anonymised)*

**Table 2:** Private providers of abortion and contraception in Cambodia

The private healthcare services relevant to abortion and contraception can be roughly divided into the following types [16, 20].	
**1. Medical consulting cabinet** managed by a doctor or a physician assistant. This facility provides general medical consultation and examination and maternal and child health services except for delivery. There is no inpatient space, and any type of surgery is not allowed.	
**2. Prenatal consulting cabinet** managed by a secondary midwife with at least three years of professional education. This facility provides antenatal care, health education, and pre- and post-delivery vaccinations. Deliveries are not conducted here.	
**3. Clinic or polyclinic** managed by a doctor with at least 5 years of experience who is either retired, has suspended their state service without pay, or has terminated their state service. Such clinics provide examination and consultation of outpatients and inpatients, conduct paramedical analysis, minor and major surgery, vaccinations, health education, maternal healthcare services including delivery and transfusion.	
**4. Pharmacy** selling various medications, including medical abortion pills.	
**5. Factory infirmary** managed by a doctor and one or two nurses. Factories are obliged to have the infirmary under their human resource department to provide basic medical care for general mild health problems.	

*Images show the area surrounding garment factories (left and middle) and a bed in a cabinet (right)*

### Study population and recruitment process

Private providers were selected as study sites since they are the preferred option of abortion for female garment factory workers in Cambodia [4]. We recruited women seeking abortion services from three private facilities: two medical consulting cabinets and one clinic. One provider had previously worked with Marie Stopes International Cambodia on a project for contraception capacity building (not for abortion services) and the others were directly asked, and agreed, to join the research. The chief or staff of the facility asked women aged ≥18, purchasing medical abortion pills or asking for a surgical abortion, if they were interested in participation. Interested women were introduced to a local researcher working for Marie Stopes International Cambodia who then explained the reasons for the study and the procedure for an interview. When a woman agreed to participate, she was asked for consent by written signature or thumbprint and her telephone number was requested for a follow-up call. Women could end the interview at any time. To protect the privacy and confidentiality of women, we secured a closed private space for the interviews and anonymized all potentially identifying information. Women were given three to four US dollars for their travel expenses and a small towel as a gift.

Additionally, we recruited 13 providers for interviews from two clinics, five medical consulting cabinets, one pharmacy, and three factory infirmaries. Three providers were working at facilities where we recruited women seeking abortion services. Other providers were purposefully selected to involve a variety of types of facilities and providers.

### Data collection methods

We developed topic guides (Appendix A and B) and modified them throughout data collection. The interviewers attended a one-day training on qualitative interviews focusing on ethics, obtaining consent from participants, and how to discuss sensitive topics. The less experienced interviewers were always accompanied by the local researcher, who also acted as an interpreter, and were shadowed by either the principle investigator and co-principle investigator for the first interviews. The interviewing teams conducted regular de-briefing after the interviews to discuss the findings as well as the interview experience. The interviews were conducted with simultaneous Khmer language translation by the local researcher who is native in Khmer and proficient in English. The interviews typically lasted 45 to 60 min. All researchers, particularly the local one, spent periods of time around the garment factories to understand the context and made notes of these observations. Interviews were recorded if women consented, transcribed in Khmer and translated into English by a Cambodian research assistant. The local researcher made follow-up calls two and 4 weeks later to women who used medical abortion to ask if the abortion was completed, if they made a follow-up visit, and to inquire about their use or plans for post-abortion contraception. Researchers also reviewed the abortion products, procedures and costs in each clinic in discussion with providers.

### Data analysis

Research members individually conducted a thematic analysis of the transcribed interviews using Dedoose software [21]. Two researchers (CM, MM) used predetermined codes from the medical abortion checklist in the Cambodian national protocol for CAC [9]. Two other researchers used a ground-up coding approach (EO, AS) and applied the developed coding frame with themes and subthemes to all interviews. The predetermined and ground-up codes were combined for comprehensiveness. The findings were then organized following a number of the headings from the national protocol on CAC, in particular the example of observation checklist for medical abortion (7 P148) to show qualitative aspects of abortion care that might not surface in quantitative and survey-style research. We did not ask participants to provide feedback on the findings.

## Results

### Description of participants

Of 24 women seeking abortion who were interested in participation, 16 were interviewed. Eight women initially said to their provider that they wanted to participate but were later busy or had returned to their hometown. Of the 16 interviewees, we able to make follow-up calls with seven. Nine interviewees were not followed up because three had a surgical abortion after medical abortion, three did not provide a working phone number, two did not respond, and one had no time. Twelve women said that this was their first abortion, three their second, and one her fourth. Fifteen women came to Phnom Penh from rural areas of Cambodia. The ages of the women ranged from 21 to 36. Thirteen said that they were married, two divorced, and one was single in a relationship. Among the married women, two lived separately from their husbands. Fourteen women already had children, and the age of their last child ranged from eight months to three years. Twelve women had left children with family in their hometowns. All 13 private providers who we asked to participate in the interview took part: three doctors, four midwives, four nurses, one pharmacist, and one self-employed without a medical background. Nine of the providers were women.

### Theme 1: factors influencing abortion-services sought and obtained by women

Theme 1 describes the findings related to abortion seeking behaviour. ‘M’ is used for women and ‘P’ for providers.

#### Sub-theme 1–1. Reasons and decision-making for abortion

Birth-spacing and financial constraints were the main reported reasons for abortion. All thirteen women who were married or in a relationship mentioned they made a joint decision with their husbands or partner and family.

*M11: I wish to have one more but not now … may be at the next two years …*

*M12: I think that I don’t have much money and now have to pay for my daughter’s milk … I made the decision together with my husband …*

*M24: After tested, I discussed with my husband whether I have to keep the baby or not … because it is not the good time for us to have the baby as our living condition is difficult and we are so young … so we decided to have abortion.*

#### Sub-theme 1–2. Information sources of abortion

Women generally asked their peers, close co-workers, older family members, or relatives for information and experiences of abortion; in particular, whether to have medical or surgical abortion, and where. Hearing about other women’s experiences strongly shaped the expectations, positively or negatively. Only two women said that they never sought information but went directly to the clinic. No woman said that they searched the internet for abortion information, though smartphones are widely used among factory workers.

*M08: My friend who experienced abortion told me about abortion … I asked her what and where she did, and she took me to here …*

#### Sub-theme 1–3. Available abortion methods

Medical abortion was available at all study sites, except for factory infirmaries, which by law do not provide this service. Based on provider reports and researcher observations, pharmacies, clinics and cabinets had one or more of three pills: Medabon, Mifeso, and a ‘Chinese pill’ (Zizhu Pharmaceutical). Medabon and Mifeso follow the World Health Organization approved regimen which is 200 mg mifepristone and 800 μg misoprostol (4 tablets of 200 μg) [22]. The regimen of the ‘Chinese pill’ is two tablets of mifepristone (25 mg per tablet) for 3 days and three tablets of misoprostol (0.2 mg per tablet) on the third day. Manual Vacuum Aspiration (MVA), was available at some clinics and cabinets.

One provider reported that women typically come to have abortion on Friday or Saturday, as the process would take place during the weekend and women could return to work on Monday to avoid losing income.

#### Sub-theme 1–4. Cost for abortion

Women reported that Medabon or Mifeso cost between 12 and 20 US dollars (USD), sometimes with an additional charge of 5 to 30 USD for vitamin injections. MVA was provided for 60 to 200 USD. One woman reported paying 200 USD, including both medical and surgical abortion, but was satisfied with the quality of services.

*M21: I feel ok … not so painful … strong enough after got service here … I thought 200 USD is not important if I am better like this …*

To put these amounts in context, the minimum monthly wage of a factory worker in Cambodia is USD170 [23].

#### Sub-theme 1–5. Selection of abortion methods

Half of the women received MVA, for the following reasons: more than 9 weeks pregnant, incomplete abortion, health care providers’ advice or it was automatically included in the abortion service. Some women preferred MVA because they thought that it was cleaner and safer than medical abortion, or they did not want to come back for a follow-up or in the case of complications. Few women considered the risk of MVA as an invasive procedure and most women were afraid that medical abortion would result in an incomplete abortion.

*M07: The provider said it is better to use vacuum aspiration...For medical abortion, I might have some difficulties when I face some problem after using it … it will be difficult to come back again if it doesn’t work well …*

### Theme 2: Women’s experiences of abortion mapped against the CAC observation checklist for medical abortion

Abortion provider in Cambodia should adhere to the national protocol for CAC. Theme 2 describes women’s experiences alongside the standards set by CAC.

#### Sub-theme 2–1. CAC task 1 – “Greets woman with respect and kindness, helps her feel comfortable and ensures privacy”

Women reported that privacy was an important factor in selecting a provider; for instance, they preferred larger providers because they afraid smaller ones may not be able to maintain confidentiality and would be less safe. Some younger and unmarried women sought a provider away from their living or working area, so that they would not encounter anyone they knew. Providers said that women never openly asked for an abortion, rather, the subject was broached indirectly.

*P05: They just ask for a pregnancy test … and they added that “after I did the pregnancy test, may I see you again …*”.

#### Sub-theme 2–2. CAC task 2 – “Assesses the woman’s health: medical and reproductive history, estimates gestation based on date of last menstruation period, assess vital signs”

In this study, all women who used medical abortion were less than 9 weeks pregnant. Most women were aware of their menstrual cycle and confirmed the pregnancy immediately after they missed their period or felt morning sickness. The most common way to estimate gestational age was ultrasound, but some declined due to the cost. Four women were asked their Last Menstruation Period (LMP). No woman had a pelvic examination.

*M13: I didn’t do ultrasound … because I missed period about 10 days … he (provider) asked me to do ultrasound … but I rejected as I didn’t want to spend lots of money ($5 USD).*

One provider said that some clients who were over 2 months pregnant came to purchase medical abortion whilst pretending to be less than 2 months pregnant, or asked someone else to buy the drugs.

#### Sub-theme 2–3. CAC task 3 – “Discuss reproductive goals, including pregnancy and family planning options”

When women came to providers, most had already decided to have an abortion following discussion with their husband or family, and the decision was respected by the providers. One woman, however, told us that:

*M24: The provider said I should have a baby now because I am young and have more energy now … she wants me to keep the baby rather than to have abortion …*

This was the only case of possible provider bias that we encountered.

#### Sub-theme 2–4. CAC task 4 – “Provide detailed information about medication-abortion side effects, warning signs, required visits and action in the event of failure of procedure”

Some women reported knowing side effects of medical abortion such as fever, exhaustion, diarrhoea, vomiting, and pain; and they received that information from providers, friends, or relatives. Most women knew that they would bleed for a few days after inserting misoprostol and needed to contact providers in case of severe bleeding. Two women experienced heavy bleeding and both visited a provider.

*M19(Note): she returned to doctor on next day of inserting pills because a lot of black bleeding and much pain, used sanitary pad every hour, was very exhausted and could not eat well.*

Infirmary workers encountered factory workers who came to the infirmary with serious abdominal pain, possibly caused by an incomplete abortion. Those women tried to be discreet and asked for painkillers for menstruation, although the bleeding was heavier than a normal menstruation.

#### Sub-theme 2–5. CAC task 5 – “Explains all aspects of the medication administration regimen, including pain management”

Most women using medical abortion were told to insert misoprostol the day after taking mifepristone. A few women took mifepristone and misoprostol on the same day, with the provider helping to insert the misoprostol. A booklet with instructions, side effects, and warning signs was available at some facilities, but some women preferred not to take any written materials for fear that other people would find out they had an abortion. Four women were provided with pain medication.

#### Sub-theme 2–6. CAC task 6 – “Provides emergency contact information in case the woman has questions or needs care”

Most providers gave their phone number to women and told them to call if they had a problem or needed more information. Some women returned or called providers if they were afraid of bleeding or pain.

#### Sub-theme 2–7. CAC task 9 – “Schedules follow-up visit to confirm completion of the medication abortion”

Most women did not return for follow-up if they did not encounter a problem. Some women who used medical abortion asked providers for a surgical abortion to ensure completion of abortion.

*P04: Clients came here seeking for cleaning (surgical abortion) to make sure if it is completed since they just saw bleeding without anything.*

Only a few women knew the signs of completion of medical abortion, and their knowledge was based on their previous abortion or miscarriage.

*M13: I noticed it had serious bleeding and it came out along with tissue … as remembered, when there is a miscarriage there are also coagulated blood come out and the size is about the size of a toe …*

#### Sub-theme 2–8. CAC task 8 – “Ensures contraceptive counselling and a method are provided if requested”

Not all women received contraceptive counselling after abortion. Some said they were informed about pills, injection, and intrauterine device (IUD), and a few women reported being advised to use traditional methods such as the calendar method. Women sought information from family members, friends, or elders in their community. Some women received contraceptive counselling at the health centre in their province after giving birth.

Of the seven women who could be followed up after abortion, three started to use modern contraception; one each of the pill, injection, and IUD. Two reasons given for not using modern contraception after abortion were still feeling weak or infrequent sex due to separation from partner.

*M07: No, I have not used any contraception yet...I thought my health is still weak...we are not too often having sex...*

Some women said they continued using traditional methods since they were afraid of infertility caused by using modern contraception, particularly by pills.

*M24: I have learnt that one of my neighbours who used pills had uterus problem … when she wanted to have a baby, she could not have a baby for about ten years …*

Women’s’ partners sometimes objected to using modern contraception because they thought that it may negatively affect their health or cause uterus problems.

*M24: Actually, my husband uses the natural method (withdrawal) … and he doesn’t want me to use any other methods because we’ve just newly married … he is worried that it affects my health....*

## Discussion

Most women seeking abortion in this study were married and had children, suggesting unmet need for contraception among female factory workers who already have children. This aligns with the data from the Cambodia demographic survey [2]. The main reasons for abortion in this study were birth spacing and financial constraints, which are same with the most common reasons for having an abortion in other countries [24]. Our findings further highlight the socio-economic context and factory work affecting the decision, such as working long hours away from their families and insufficient financial security.

Ultrasound was the most common method to determine the gestational age in our study, but some women declined it due to the cost. Gestational age can be accurately estimated based on last menstrual period and physical examination together [6, 22], therefore providers should be able to estimate the gestational age without using ultrasound. The CAC protocol rightly emphasizes the importance of clear, comprehensive, and sensitive communication with women seeking abortion services [9]. There was only one report of possible provider bias when it was recommended that the woman should continue with the pregnancy. Although we do not know how widespread this attitude is, some qualitative studies reported that provider’s attitudes and values to abortion strongly affect the availability of abortion services [25, 26]. This possible provider bias in Cambodia could impede younger and women without a child from accessing safe abortion services. The National Strategy for Reproductive and Sexual Health in Cambodia 2017–2020 identifies “information” as a key area to address in order to continue making progress to strengthen safe abortion services [27]. However, it was difficult to clarify whether providers gave accurate and adequate information to women to decide on an abortion methods in our study. Half of the women underwent MVA, as it was viewed as a safer and quicker option. This preference for MVA is similar with findings in other papers [8, 28], but women in our study were not always sufficiently informed about the pros and cons of both methods. The desire to complete the abortion during weekends to minimise time off work was an important factor for factory workers in deciding the timing and methods of abortion. Reluctance to attend follow-up appointments was another reasons for choosing MVA. Similarly, detailed instruction and the process of medical abortion were not well explained. Many women did not know the signs of abortion completion and did not have enough confidence in dealing with bleeding and pain. As a result, some women returned to the provider for a surgical abortion. Only three women started post abortion family planning. Many women expressed fear and hesitation to use modern contraception as reported in previous papers [5, 29, 30], with some reporting a belief that contraceptive pills make women infertile. Most of the interviewees knew about modern contraceptive methods, which is in line with the result of a national survey among the general population and a survey among factory workers [2, 4]. However, contraception knowledge was mostly obtained from family or friends. In addition to concerns about side-effects, the infrequent sex due to separation from husbands affected the decision not to use contraception, which is consistent with a previous survey among factory workers [4].

Based on the findings from this study, we recommend two main areas for future work. The first is regarding provision of accurate and adequate information about abortion methods and modern contraception. Provision of detailed information about medical abortion procedure can be improved to empower women to manage the process by themselves. WHO’s guideline recognises that women can play an essential role in the use of medical abortion, under certain conditions in which access to appropriate information and back-up health care facilities are ensured [31]. A previous study reported that effective counselling can offer women a sense of confidence by helping them prepare for possible side effects and complications [32]. A booklet with instructions was provided to woman using medical abortion. However, it was perceived to be indiscreet and few women took it, as has been reported elsewhere [32]. Therefore alternative means should be considered and future work needs to explore effective ways to deliver appropriate information privately and refer women to safe providers, such as via call-centres or social media. There is a need for reliable information on modern contraception, which can be delivered by qualified healthcare providers through in-person and alternative methods. The quality of family planning counselling should be improved to clarify the any misconceptions surrounding modern contraception and to focus on women’s actual lifestyle and needs. In response to the findings of this study, we developed short videos on modern contraceptive methods and how to take medical abortion. The videos are available on the Facebook page of MSI Cambodia, and an evaluation of their impact will be reported in a separate paper. Second, there is a need to increase the opportunity to provide family planning counselling and services for married women with children. In our study most women with unmet need for family planning in Cambodia already had a child suggesting that services for married women with children should be strengthened. Women with children have several opportunities to visit health facilities for antenatal care, delivery, postnatal care, and immunizations, therefore health care providers should utilise these opportunities to provide family planning services. A study in the Philippines found that most women who wish to delay pregnancy missed the opportunity to receive family planning counselling during their public facility visits [33]. Improving the availability and quality of family planning counselling at health facilities can increase the use of contraception and reduce unintended pregnancies among women with children.

This study has some strengths and limitations. A strength of this study derives from our thorough knowledge of the Cambodian garment factory setting and the multi-disciplinary approach. The interviewers spent periods of time around the garment factories, which helped us to understand the living condition of factory workers and be sensitive to the local culture. Limitations of this study include that the sensitive nature of the research topic might have influenced the woman’s decision to participate in the interview and the responses that they gave. Most of our interviewees were married. Single or unmarried women might have hesitated to participate since pregnancy outside of marriage is not socially acceptable in Cambodia [2]. In addition, characteristics of researchers such as their gender and nationality might have influenced interaction with research participants. We tried to mitigate this by alternating the foreign researchers conducting the interviews, while always having a local researcher present as translator and facilitator, and by triangulating with secondary sources and other material from our NGO partner. Some women who knew the local Marie Stopes clinic might have trusted the interviewers and may have been more inclined to participate or talk about their concerns to interviewers. We did not interview women attending public healthcare services, because garment factory workers typically seek abortions at private healthcare services which are perceived as “friendly, confidential and clean” [4, 29].

## Conclusion

In conclusion, this paper described the factors influencing on seeking the abortion services and abortion experiences among women working in garment factories. While abortion and contraception services are widespread and affordable, not all women are receiving comprehensive abortion care and contraceptive counselling. Future work to address this gap could involve the development of appropriate interventions and informative tools for women in the Cambodian garment industry such as through existing client contact-centres or social media, including creation of videos or posts on topics that come from clients questions.

## Data Availability

The data that support the findings of this study are available on request from the corresponding author [CS]. The data are not publicly available because they contain information that could compromise research participants’ privacy and consent.

## Interview guide for women seeking MA

### Background information

**Table:**

What is your age?	
Where are you from?	
How long have you been in Phnom Penh?	
Where do you work and what is your role? (Don’t need specifics of factory)	
How is your work?	

### Relationships and living situation

**Table:**

Are you married?	
If no: do you have a relationship?	
Do you have children?	
How many?	
How old are they?	
Where do they live?	
What sort of accommodation do you live in?	
Who do you live with?	

### MA decision-making, expectations and information seeking

**Table:**

Have you had an abortion before?	
When did you realise you were pregnant this time?	
How did you realise you were pregnant?	
What happened after that? Feelings Further confirmation sought Who discussed it with Why decided to have an abortion this time Do you think this is a common reason for women to have abortion here?	
How did you decide what to do? Did you talk to your family or friends? How did you make the decision?	
Why did you choose this provider?	
How did you learn about MA? Do you have any friends or family who have had MA? What was their experience?	
Why did you choose MA instead of another way?	

*If have not taken MA yet:*

**Table:**

Did you come with anyone today?	
When do you think you will take the MA?	
What do you expect to happen when you take the MA?	
Have you been told will happen? Who has told you this? What has the provider said?	
How will you manage it?	
What will you do if you need more advice?	
Will you need to take any time off work?	
What are your feelings about taking it?	

*If have taken MA already:*

**Table:**

Did you come with anyone to get the MA?	
When did you take the MA?	
What happened next? If any decisions made or further care sought: ask about these and why	
How did you manage the bleeding? Did you use any products? How much did these cost?	
Did you take any time off work?	
Was the MA process what you expected? What did the provider tell you? Did anyone else tell you what to expect?	
What were your feelings about taking MA?	
Did anyone help you at home?	

### Attitudes and barriers around MA

**Table:**

Was it easy or hard to come to buy MA? Why was it easy/hard? What would have made it better for you?	
Who would you tell and who would you not tell? Would you tell your friends?	
Do people here think abortion is a bad thing? Who says this?	

*If have not taken MA yet:*

**Table:**

Do you know how much the cost is? Will anyone help you pay?	
Did you have to pay for transport here?	

*If have taken MA already:*

**Table:**

What were the biggest challenges to taking MA?	
How much did the MA (/MVA) cost? Did anyone help you pay? Did you have to pay for anything else? Transport?	

### Contraception

**Table:**

Have you used contraception before?	
If yes: which methods? How long for? What was your experience of it? Why did you stop using it?	
If no: why do you not use contraception?	
Do you plan to use any contraception after the abortion?	
*If yes:* do you know which method you will have? Do you know when you will get it? Do you know where? *If reported side effects:* do you know what you will do if you get the side effects?	
*If no:* why not? How will you prevent pregnancy? Do you know which methods are available?	
How do you make decisions about FP? Where would you get information about FP? Have you ever seen any videos or information online about FP? Do you and your friends ever talk about contraception?What contraception do your friends use? What do they say about it?	

### General life questions

**Table:**

What are your plans for the future with your family and your work?	
Do you have a mobile phone? What type is it? What do you use it for?	
How do you enjoy yourself on your day off?	

**Is there anything you want to ask us?**

## Interview guide for private providers

### MA provision

**Table:**

How long they have been providing MA	
Available types of drug for MA	
Approximate number of clients per week	
Information given to clients	

### Training

**Table:**

What formal training they have been given	
How they keep up to date	
What support they would like	

### Post-abortion care and support

**Table:**

Post-abortion issues encountered	
Post-abortion care provided	
Suggestions for interventions	

## Abbreviations

CAC: Comprehensive abortion care
MVA: Manual vacuum aspiration
IUD: Intrauterine device
LMP: Last menstruation period
